# A systematic review of OCT and OCT angiography in retinal vasculitis

**DOI:** 10.1186/s12348-023-00327-4

**Published:** 2023-01-30

**Authors:** Dhanach Dhirachaikulpanich, Kanat Chanthongdee, Yalin Zheng, Nicholas A. V. Beare

**Affiliations:** 1 Department of Eye and Vision Sciences, University of Liverpool, Liverpool, UK; 2 Faculty of Medicine, Siriraj Hospital, Mahidol University, Bangkok, Thailand; 3 Department of Physiology, Faculty of Medicine Siriraj Hospital, Mahidol University, Bangkok, Thailand; 4 Liverpool Centre for Cardiovascular Science, University of Liverpool and Liverpool Heart and Chest Hospital, Liverpool, UK; 5 St Paul’s Eye Unit, Liverpool University Hospitals NHS Trust, Liverpool, UK

**Keywords:** Retinal vasculitis, Uveitis, OCT, Optical coherence tomography, OCT-Angiography, OCT-EDI

## Abstract

**Background:**

Retinal vasculitis is a component of uveitis for which the Standardisation of Uveitis Nomenclature (SUN) working group has no standard diagnostic criteria or severity grading. Fluorescein angiography is the gold standard test to assess retinal vasculitis, but is invasive and time-consuming. Optical coherence tomography (OCT) provides non-invasive detailed imaging of retinal structures and abnormalities, including blood vessel architecture and flow with OCT angiography (OCT-A). However, use of OCT in retinal vasculitis beyond assessing macular oedema, is not well established. We conducted a systematic review to understand the features of retinal vasculitis in OCT, Enhanced-depth imaging OCT (OCT-EDI) and OCT-A imaging.

**Methods:**

The systematic search was done in March 2022 and updated in January 2023, through PubMed, EMBASE and the Web of Science database for studies related to OCT, OCT-EDI and OCT-A findings and retinal vasculitis. Bias assessment was assessed using JBI Critical Appraisal Checklist, and any findings associated with retinal vasculitis were extracted by qualitative analysis.

**Results:**

We identified 20 studies, including 8 articles on OCT, 6 on OCT-EDI and 6 on OCT-A. The studies included analytical retrospective studies, case-series, and a case–control study. Five OCT studies reported secondary complications could be detected, and four reported retinal thickness alteration in retinal vasculitis. Five studies explored choroidal thickness alteration in OCT-EDI, and four explored capillary density alterations in retinal vasculitis using OCT-A. The heterogeneity in the studies’ analysis and design precluded a meta-analysis.

**Discussion:**

There were no clear OCT, OCT-EDI or OCT-A findings that demonstrated potential to supersede fluorescein angiography for assessing retinal vasculitis. Some signs of macular structural effects secondary to retinal vasculitis may help prognostication for vision. The OCT signs of inflamed retinal vessels and perivascular tissue is an unexplored area.

**Supplementary Information:**

The online version contains supplementary material available at 10.1186/s12348-023-00327-4.

## Introduction

Retinal vasculitis is one of the presentations of posterior uveitis [1]. The causes of retinal vasculitis are heterogenous ranging from infections such as tuberculosis [2] to systemic inflammatory diseases such as Behcet’s disease, systemic lupus erythematosus (SLE) and sarcoidosis [3, 4]. Retinal vasculitis can also be drug-induced or idiopathic [5]. Assessing retinal vasculitis can be challenging. Fundoscopic findings may be minimal, non-specific or not differentiate active disease. Fluorescein angiography, preferably wide-field, is needed to determine the presence of active vasculitis and its severity. However, there is no consensus accepted classification or grading system for retinal vasculitis [6–8]. Fundoscopic findings include, vascular sheathing or classical tram line appearance [9], perivascular retinal infiltrates, perivascular haemorrhages, retinal vessel occlusion, vessel beading and neovascularisation secondary to ischaemia. On fluorescein angiography [10–12], retinal vasculitis shows breakdown of the inner blood-retina barrier (vascular leakage) or occlusion of vessels. Leakage can be segmental or widespread, affect any vessel and is widely seen as a marker of inflammatory activity [4]. Occlusion can be in the form of capillary non-perfusion or of larger vessels and may be due to previous vasculitis which is currently inactive. Staining of vessel walls can also be seen in active and inactive disease. Fluorescein angiography is time-consuming and invasive as it requires intravenous dye injection [7, 13].

**Fig. 1:**
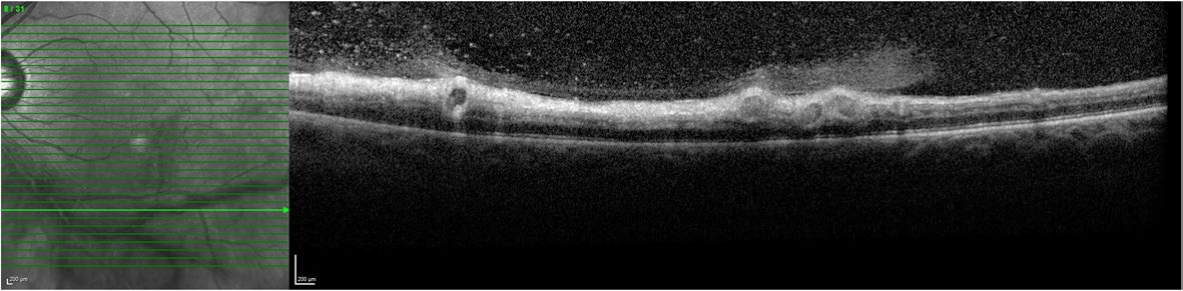
OCT of a patient with active retinal vasculitis in Behcet’s Disease showing enlarged retinal vessels with hyperreflective walls, hyperreflective lumens and adjacent vitritis

Optical coherence tomography (OCT) is a non-invasive imaging modality that shows details of retinal structures and abnormalities [14, 15]. Retinal pathology associated with posterior uveitis can easily be observed using OCT, such as changes in retinal thickness, disruption of retinal layers and macular oedema [16, 17]. OCT of inflamed retinal vessels can identify enlarged vessels, hyperreflective vessel walls, hyperreflective lumen, inflammatory material in the adjacent vitreous and thickened perivascular retina (Fig. 1). Enhanced-depth imaging OCT (OCT-EDI) better visualises choroidal structures, [18] and changes in choroidal thickness can relate to posterior uveitis [19, 20]. OCT angiography (OCT-A), non-invasively images perfused retinal and choroidal vessels of the central macula [21, 22]. OCT-A can identify vessel closure and non-perfusion but not blood-retina barrier breakdown.

Previous non-systematic reviews have examined multimodal imaging in uveitis and retinal vasculitis but OCT modalities in retinal vasculitis has not been reviewed [7, 13, 23, 24]. Therefore, we initiated this systematic review to understand the findings in OCT, OCT-EDI and OCT-A associated with retinal vasculitis.

## Methods

### Search strategies and data sources

This systematic review was done following the guidelines of PRISMA 2020 [25]. This study was a systematic review of previous studies without new intervention; hence, ethical approval was not required.

A systematic electronic search of Pubmed/Medline, Web of Science and Embase was done in March 2022 and updated in January 2023. The following search terms were used “oct” OR “optical coherence tomography” AND “retinal vasculitis”. Duplication in the included papers was removed using Endnote’s deduplicate and manual removal by the authors, DD and KC.

### Study selection

Eligible studies met following criteria: (1) the study was on patients diagnosed with retinal vasculitis (2) the diagnosis of retinal vasculitis was based on medical records and/or fluorescein angiography imaging (3) the study reported features of retinal vasculitis on OCT or OCT-EDI or OCT-A (4) case–control, cross-sectional, prevalence or case-series studies were included (5) the study was a peer-reviewed article. Studies were excluded if (1) it was a review, (2) the article was not in English (3) a case report/case series which reported less than 5 patients. Two authors, DD and KC, independently conducted a study selection and exclusion. Discordance was resolved by the senior author, NAVB.

### Data extraction and bias assessment

Two authors, DD and NAVB, collaboratively performed bias evaluation and did data extraction. The following data were tabulated and extracted: Study site and year of publication, imaging modalities, age of participants (mean and range), number of participants with retinal vasculitis, sex, the causative disease of retinal vasculitis, retinal vasculitis diagnostic criteria of the study and the study type.

Bias assessment was conducted using JBI Critical Appraisal Checklist for analytical cross-sectional studies, prevalence studies, case–control studies or case series, depending on the type of each study. The included studies were used in the data synthesis [26, 27]. Studies were only included if they scored more than half of each JBI Critical Appraisal Checklist.

### Data synthesis

It was not possible to perform quantitative analysis or meta-analysis due to each associated publication’s different nature and parameters. For qualitative analysis, the main OCT, OCT-EDI or OCT-A findings associated with retinal vasculitis were descriptively reported.

## Results

### Searching results and the included studies’ characteristics

**Fig. 2:**
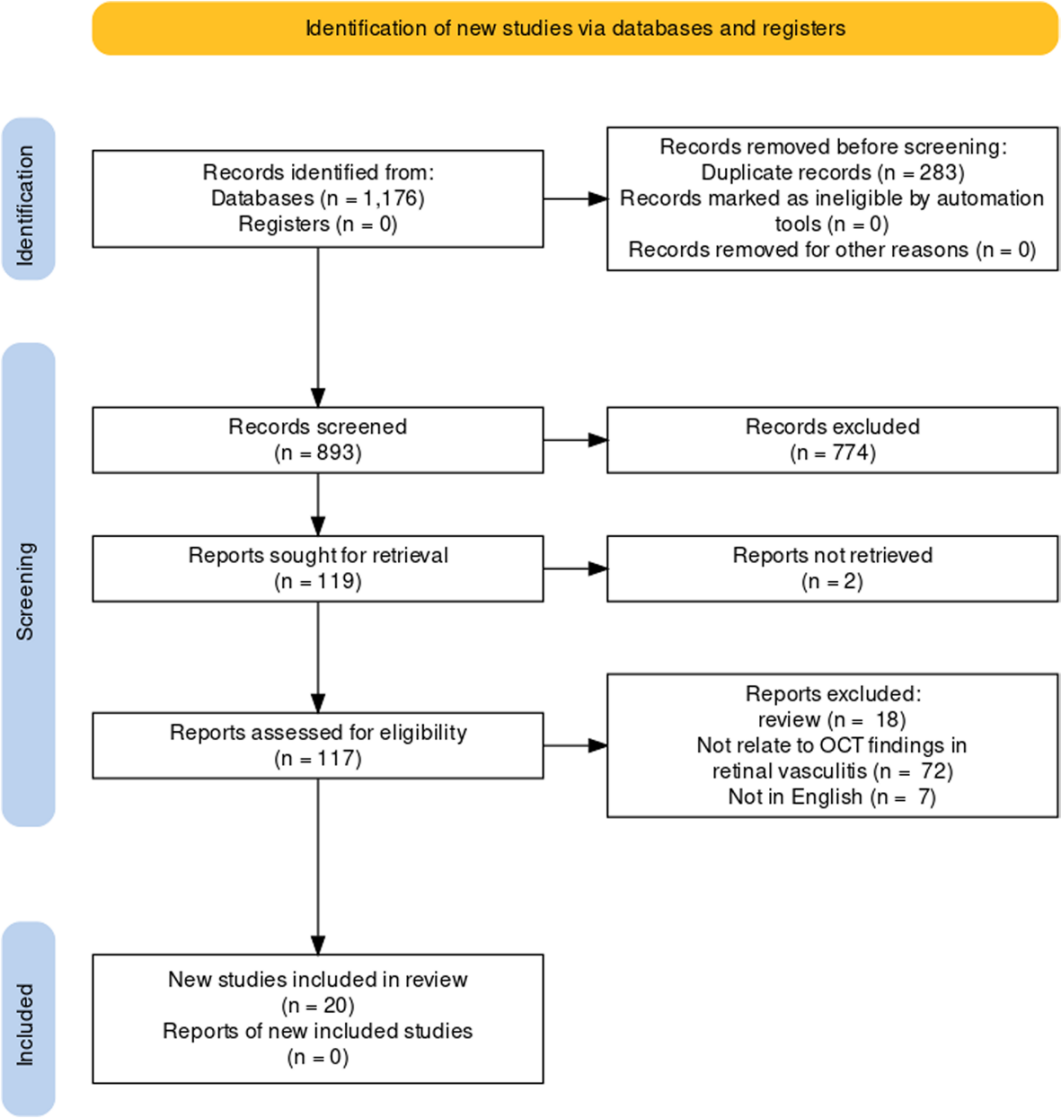
PRISMA flow diagram for study selection

Figure 2 summarises the search methodology following PRISMA2020 guidance. We retrieved a total of 800 articles from Pubmed/MEDLINE (*n* = 424), EMBASE (*n* = 580) and the Web of Science databases (*n* = 172). We removed duplicate articles using an automatic function in Endnote X9 programs and also by manual detection. Of the 893 remaining articles, 774 were excluded by initial screening of the title and abstract. One hundred and nineteen articles remained for the full-length article review and 99 were excluded. Finally, 20 articles were included in this systematic review.

The study types included 13 analytical cross-sectional studies [28–40], 6 case series [41–46] and 1 case–control study [47]. The characteristics of all included studies are shown in Table 1. Eight articles [28–30, 38, 40–43] reported features of retinal vasculitis on OCT, 6 on OCT-EDI [31–33, 37, 44, 47] and 6 on OCT-A [34–36, 39, 45, 46]. The underlying diagnosis of uveitis reported in the included studies included idiopathic retinal vasculitis, Birdshot Chorioretinopathy, Eales disease, sarcoidosis, tuberculosis, rheumatoid arthritis, multiple sclerosis, SLE, acute retinal necrosis, Behcet’s Disease, c-ANCA related systemic vasculitis, HIV, Toxoplasmosis, Takayasu arteritis, Rickettsial infection, idiopathic retinal vasculitis aneurysms and neuroretinitis syndrome, west Nile virus infection, cytomegalovirus retinitis, endogenous endophthalmitis, Susac syndrome, measles, poststreptococcal uveitis, Toxocariasis, pars planitis, intraocular lymphoma, syphilis and Crohn’s disease.

**Table 1:** Characteristics of included studies

Study	Country	Age of participants in diseases group (range(mean), years)	Image modality	Number of participants in diseases group	Sex (female: male)	Disease	Criteria for the diagnosis of retinal vasculitis	Study type	JBI Critical Appraisal Checklist score
Monnet D et al., 2007^a^ [28]	France	21–79 (median = 55.9)	OCT	80	51:29	Birdshot Chorioretinopathy	previous medical record, clinical examination at baseline, and FA imaging(scoring)	analytical cross-sectional study	8/8
Karampelas M et al., 2015 [29]	UK	19–85 (median = 45)	OCT	82	37:45	idiopathic, sarcoidosis, tuberculosis, rheumatoid arthritis, multiple sclerosis, SLE, acute retinal necrosis, Bechet, cANCA, HIV	previous medical record and FA imaging(scoring)	analytical cross-sectional study	6/8
Maleki A et al., 2016 [30]	USA	7–83(42.7)	OCT	80	55:25	idiopathic	previous medical record, clinical examination at baseline, and FA imaging	analytical cross-sectional study	8/8
Teussink MM et al., 2016 [41]	Netherlands	25–76 (51)	OCT	21	9:12	Birdshot Chorioretinopathy	previous medical record and FA imaging	case series	10/10
Goel N et al., 2018 [42]	India	16–54(27.97)	OCT	66	4:62	Eales disease	previous medical record, clinical examination at baseline, and FA imaging	case series	8/10
Knickelbein JE et al., 2018 [43]	USA	38–64(55.5)	OCT	11	9:2	Birdshot Chorioretinopathy	previous medical record and FA imaging	case series	10/10
Zarei M et al., 2021 [38]	Iran	18–48 (31.6)	OCT	15	4:11	Behcet’s Disease	previous medical record, clinical examination at baseline, FA imaging (scoring)	analytical cross-sectional study	6/8
Silpa-Archa S et al., 2022 [40]	Thailand	15–73 (40)	OCT	48	28:20	retinal vasculitis with positive QuantiFERONTB Gold Test	previous medical record, clinical examination at baseline, FA imaging	analytical cross-sectional study	8/8
Birnbaum AD et al., 2014 [44]	USA	44–68 (58)	OCT-EDI	14	12:2	Birdshot Chorioretinopathy	previous medical record, clinical examination at baseline, and FA imaging (scoring)	case series	9/10
Böni C et al., 2016 [31]	USA	(57.3)	OCT-EDI	76	54:32	Birdshot Chorioretinopathy	previous medical record	analytical cross-sectional study	8/8
Onal S et al., 2018 [32]	Turkey	18–46 (29.75)	OCT-EDI	28	9:19	Behcet’s Disease	previous medical record, clinical examination at baseline, FA imaging (scoring)	analytical cross-sectional study	6/8
Shirahama S et al., 2019 [33]	Japan	(45)	OCT-EDI	30	10:20	Behcet’s Disease	previous medical record and FA imaging	analytical cross-sectional study	6/8
Bousquet E et al., 2021^a^ [37]	France	(60)	OCT-EDI	80	48:32	Birdshot Chorioretinopathy	previous medical record and clinical examination at baseline	analytical cross-sectional study	8/8
Kumar A et al., 2021 [47]	India	(34.61)	OCT-EDI	23	3:20	idiopathic	previous medical record, clinical examination at baseline, and FA imaging	case–control study	8/10
Abucham-Neto JZ et al., 2018 [45]	Brazil	24–67 (36)	OCTA	10	7:3	Behcet’s Disease, Toxoplasmosis, Sarcoidosis, Takayasu arteritis, idiopathic	previous medical record and FA imaging	case series	6/10
Tian M et al., 2019^b^ [34]	Switzerland	(45.9)	OCTA	58	32:26	IU (sarcoidosis, Behcet, Idiopathic)	previous medical record, clinical examination at baseline, FA imaging	analytical cross-sectional study	8/8
Tian M et al., 2019^b^ [35]	Switzerland	(45.5)	OCTA	88	54:34	IU (sarcoidosis, latent TB, Behcet’s Disease, Idiopathic)	previous medical record, clinical examination at baseline, FA imaging	analytical cross-sectional study	8/8
Emre S et al., 2019 [36]	Turkey	(39.44)	OCTA	16	10:6	Behcet’s Disease	previous medical record, clinical examination at baseline, and FA imaging	analytical cross-sectional study	6/8
Abroug N et al., 2021 [39]	Tunisia	No data	OCTA	17	No data	Birdshot Chorioretinopathy, pars planitis, intraocular lymphoma, syphilis, undifferentiated cause	previous medical record and FA imaging	case series	7/10
Noori J et al., 2021 [46]	USA	(31.5)	OCTA	284	117:167	idiopathic retinal vasculitis, Eales disease, sarcoidosis, tuberculosis, Birdshot Chorioretinopathy, multiple sclerosis, SLE, acute retinal necrosis, Behcet’s Disease, Toxoplasmosis, Rickettsial infection, idiopathic retinal vasculitis aneurysms and neuroretinitis syndrome, west Nile virus infection, cytomegalovirus retinitis, endogenous endophthalmitis, Susac syndrome, measles, poststreptococcal uveitis, Toxocariasis, syphilis and Crohn’s disease	previous medical record, clinical examination at baseline, and FA imaging	analytical cross-sectional study	6/8

*FA* Fluorescein angiography

^a^, ^b^ same group of researchers

### Bias assessment

We assessed the bias using JBI Critical Appraisal Checklist for analytical cross-sectional studies, prevalence studies, case–control or case series, depending on the type of study. All eligible studies that scored on more than half of the checklist are considered appropriate to include for synthesis, as shown in Table 1 and Supplementary File 1. Among the checklist, we noticed some common biases in these eligible articles. Five of thirteen analytical cross-sectional studies [29, 33, 36, 38, 39] did not mention possible confounding factors associated with observed imaging features in OCT related to the diagnosis of retinal vasculitis and did not state how to deal with associated confounding factors. For the case series, three studies did not report appropriate demographic data [44–46].

Of all primary studies related to OCT and retinal vasculitis, there was only one diagnostic test accuracy study. This study by Tian M et al. [35] used accuracy type statistics, including sensitivity and specificity to compare OCT-A findings with fluorescein angiography leakage, as the gold standard for diagnosing retinal vasculitis.

### OCT findings

#### OCT signs of active inflammation associated with retinal vasculitis

Seven of the 8 studies reported OCT findings associated with the secondary effects of retinal vasculitis, including cystoid macular oedema, epiretinal membrane and disruption of ellipsoid zone. One study reported on perivascular retinal thickness, but none of the 8 studies [28–30, 38, 40–43] reporting OCT features related to retinal vasculitis, reported primary vascular abnormalities.

Five studies reported the association between OCT findings and secondary effects in retinal vasculitis [28, 30, 41, 42]. In the first study, Monnet D et al. conducted an analytical cross-sectional study of 80 birdshot chorioretinopathy patients (160 eyes) [28]. They graded the retinal vasculitis using fluorescein angiography, scoring from 0–4, with 4 being the most severe leakage. These leakage scores were compared with associated OCT findings, including epiretinal membranes and the third hyperreflective band (the hyperreflectivity band in the levels of photoreceptors) [48]. This third band corresponds to the junction of the inner and outer segments of the photoreceptors. The result showed that the increasing grade of fluorescein leakage was associated with the presence of epiretinal membranes (logistic regression models with the generalised estimating equation, adjusted *p*-value = 0.024) but not the intactness of third hyperreflective band (adjusted *p*-value = 0.386). They also compared the presence/absence of retinal vasculitis with the same OCT findings. The logistic regression models showed no association between the presence of retinal vasculitis and epiretinal membranes (adjusted *p*-value = 0.433) or the intactness of the third hyperreflective band (adjusted *p*-value = 0.418).

Maleki A et al. conducted an analytical retrospective study of 80 patients with idiopathic retinal vasculitis (150 eyes) [30]. Poor visual outcome was defined as visual acuity lower than 20/40 or decrease in 2 or more lines. Poor visual outcome was associated with the OCT findings of cystoid macular oedema (Fischer’s exact test, *p* = 0.0001) and epiretinal membrane (*p* = 0.008). Only cystoid macular oedema remained as an indicator after using multivariate binary logistic regression analysis to correct for confounding factors (OR 5.54, 95% CI 1.81–16.99, *p* = 0.003); other prognostic factors were logMAR visual acuity at the first visit (OR 3.78, 95% CI 1.75–8.16, *p* = 0.001) and macular ischemia (OR 5.12, 95% CI 1.12–23.04, *p* = 0.036).

Teussink MM et al. reported a narrative case series of OCT findings in birdshot chorioretinopathy in 21 patients (42 eyes) in 2016 [41], without statistical analysis. The authors noted disruption of the ellipsoid zone on OCT in 7 (33%) patients. Among these 7 patients, 4 had these disrupted ellipsoid zones reconstitution after resolution of the retinal vasculitis, whereas 3 with poorly responsive retinal vasculitis did not. The authors concluded that this ellipsoid zone disruption might relate to the activity of retinal vasculitis.

In a study published in 2018, Goel N et al. conducted case series in 79 eyes of 66 patients with Eales disease and active vasculitis [42]. The study didn’t report any association type of statistics. They indicated that OCT showed more macular complications in the eyes with active vasculitis than in the eyes with inactive vasculitis. In this study, macular complications included macular oedema, epiretinal membrane, macular thinning, hard exudates, haemorrhages, inner retinal or inner limiting membrane folds, pre-macular haemorrhages and macular hole.

Another study by Silpa-Archa S et al. reported the OCT findings in 73 eyes of 48 patients with retinal vasculitis [40]. They compared OCT findings in patients with retinal vasculitis with the poor visual outcome (visual acuity worse than 20/200) and good visual outcome (visual acuity better or equal to 20/200). The results showed a higher proportion of OCT findings, including presence of epiretinal membrane and outer retinal disruption in the poor visual outcome group (Fischer’s exact test, *p* < 0.05), but there was no difference in the proportion of cystoid macular oedema, overlying vitritis and submacular fluid between two groups. The presence of outer retinal disruption remained as an indicator of poor visual outcome after using multivariate binary logistic regression analysis to correct for confounding factors (OR 21.12, 95% CI 1.39–320.28, *p* = 0.028).

#### Macular thickness alteration associated with the retinal vasculitis

Two studies have highlighted that increased central macular thickness associated with retinal vasculitis [28, 29]. The study by Monnet D et al. of birdshot chorioretinopathy patients measured the macular thickness using OCT3 software (Zeiss-Humphrey) [28]. The program calculates the mean retinal thickness of 512 points within 1 mm of the fixation point. The study found that an increase in grading of fluorescein leakage severity was related to increased macular thickness (adjusted *p*-value < 0.001). However, there was no difference when the mean macular thickness was compared between the presence and absence of retinal vasculitis groups (adjusted *p*-value = 0.247).

Another study by Karampelas M et al. in 2015 reported an analytical cross-sectional study from 82 eyes of 82 uveitis patients [29]. The central macular thickness was calculated using Topcon’s FastMap software. The study graded the vasculitis by measuring macular and peripheral leakage index, defined as leakage area as a percentage of total area using wide-field fluorescein angiography images of the macula or the peripheral area. Their result found that central macular thickness was positively correlated with macular leakage index (Spearman correlation coefficient (*r*) = 0.485, *p*-value < 0.001) and weakly with foveal avascular zone size (*r = *0.291, *p*-value = 0.03), and not correlated with peripheral leakage index (*r* = -0.157, *p*-value = 0.248).

The perivascular retinal thickness in retinal vasculitis has been investigated by OCT. The case series by Knickelbein et al. in 2018 characterised 11 patients (22 eyes) with retinal vasculitis from birdshot chorioretinopathy by using OCT scans centred on the proximal branches of the superior and inferior retinal vessels, fovea, and the optic nerve head [43]. They measured the perivascular retinal thickness by using system software (Zeiss Cirrus Viewer) on 6 × 6 mm scans of the proximal temporal arcades. The mean perivascular retinal thickness at diagnosis (mean = 397.46 um) showed a decrease after one month of treatment with prednisolone (mean = 346.9 um; paired t-test, *p*-value < 0.00001, *n* = 3 patients). For the other 8 patients, the authors concluded that 4 patients had perivascular retinal thickness increased during active disease and decreased during quiescence (no statistics applied), while another 4 patients did not have uveitis activity or perivascular retinal thickness change.

A study by Zarei M et al. in 2021 correlated peripapillary OCT parameters and fluorescein angiography inflammatory score in 15 patients (28 eyes) with Behcet's disease and retinal vasculitis [38]. The fluorescein angiography inflammatory score was developed to indicate the inflammatory activity of each fluorescein angiography image ranging from 0 and 43. Central subfield macular thickness positively correlated with fluorescein angiography inflammatory score (Spearman correlation coefficient (*r*) = 0.413, *p*-value < 0.001). The fluorescein angiography inflammatory score also positively correlated with the peripapillary retinal thickness measured at 2.2 mm (*r* = 0.443, *p*-value < 0.001) and 3.45 mm diameter (*r* = 0.707, *p*-value < 0.001), and peripapillary retinal nerve fibre layer thickness (*r* = 0.850, *p*-value < 0.001).

### OCT-EDI findings

#### Choroidal thickness alteration as a finding associated with the retinal vasculitis

Five studies have investigated subfoveal choroidal thickness alteration in retinal vasculitis [31, 33, 37, 47]. Böni C et al. conducted an analytical cross-sectional study of 86 patients (172 eyes) with birdshot chorioretinopathy in 2016 [31]. They compared the presence of retinal vasculitis with OCT-EDI findings, including change in choroidal thickness, suprachoroidal space, macular lesion (focal or diffuse lesion) and any choroidal lesion. There was no significant association between the presence of retinal vasculitis with choroidal thickness (OR 1.43, adjusted *p*-value = 0.37), suprachoroidal space (OR 0.91, adjusted *p*-value = 0.80), macular lesion (OR 0.80, adjusted *p*-value = 0.50) and any choroidal lesion (OR 1.04, adjusted *p*-value = 0.91).

The second study by Shirahama S et al. was an analytical cross-sectional study that included 30 Behcet's uveitis patients (51 eyes) [33]. This study graded vasculitis by a fluorescein angiography leakage score for the peripheral retina, macula, and optic disc on a scale from 0 to 3 [49]. The leakage score was then compared with the subfoveal choroidal thickness measured manually with OCT-EDI. They reported a significant positive correlation between the subfoveal choroidal thickness and leakage score in the total retina (*r*^2^ = 0.210, *p* = 0.0007, linear regression analysis).

Bousquet E et al. conducted an analytical cross-sectional study of 80 birdshot chorioretinopathy patients (160 eyes) [37]. The study found that subfoveal choroidal thickness was significantly greater in birdshot chorioretinopathy patients with vasculitis than without vasculitis (274.6 um vs 228.5 um, *p*-value = 0.006, Student t-test). The presence of retinal vasculitis was associated with the choroidal vascularity index, which was defined by the ratio of the luminal area to the total choroidal area in OCT-EDI. The result showed that the choroidal vascularity index was positively associated with the presence of the retinal vasculitis in the univariate analysis (*p*-value < 0.001, generalised linear regression models with generalised estimating equation) but not the multivariate analysis (*p*-value = 0.24).

Onal S et al. reported the analytical cross-sectional study of 28 Behcet’s uveitis patients (56 eyes). The study investigated the correlation of retinal/choroidal thickness with retinal vascular leakage using fluorescein angiography scores ranging from 0 to 40 developed by the Angiographic Scoring for Uveitis Working Group [32]. The result showed there was no statistically significant correlation between the fluorescein angiographic score and the subfoveal choroidal thickness (*r* = -0.221, *p*-value = 0.105). However, the central foveal thickness was correlated with the fluorescein angiographic score (Spearman correlation coefficient (*r*) = 0.605, *p*-value < 0.001). The sub-analysis showed that retinal vascular staining/leakage score correlated with choroidal stroma to choroidal vessel lumen ratio in OCT (*r* = 0.300, *p*-value = 0.036).

Kumar A et al., in a case–control study compared 23 acute idiopathic retinal vasculitis patients (36 eyes) with 25 control patients (50 eyes) using OCT-EDI [47]. There was a significant greater subfoveal choroidal thickness in the vasculitis group compared with the control group (338.86um vs 296.72um, *p* < 0.001, Mann–Whitney U test).

#### Other OCT-EDI findings associated with the retinal vasculitis

A case-series of 14 birdshot chorioretinopathy patients (58 eyes) by Birnbaum et al., 2014 correlated the OCT-EDI findings with the retinal vasculitis using a fluorescein angiography leakage score ranged from 0 (none) to 2 [44]. The leakage score correlated with the presence of suprachoroidal fluid (Spearman correlation coefficient (*r*) = 0.45, *p*-value < 0.001). but not RPE disruption (*r* = -0.20, *p*-value = 0.15), retinal thickness (*r* = 0.01, *p*-value = 0.97) nor ellipsoid layer disruption (*r* = -0.21, *p*-value < 0.11).

### OCT-A findings

#### The primary sign of active inflammation in OCT-A

The case-series by Abucham-Neto JZ et al. examined the possibility of detecting the primary sign of active inflammation in retinal vasculitis, which they expected to see in relation to vascular sheathing in OCT-A. They explored 3 × 3 mm and 8 × 8 mm OCT-A of 10 patients (19 eyes) with retinal vasculitis [45]. They reported that they could not detect any primary sign of active inflammation around the affected vessels on OCT-A, even though 74% of eyes had shown active signs on fluorescein angiography images. However, complications, including capillary dropout, enlarged foveal avascular zone, telangiectasia, shunts and area of neovascularisation, could be detected in 74% of eyes using OCT-A. This study did not do any association type of statistical analysis.

#### Capillary vessel density alteration associated with the retinal vasculitis

Four studies have reported retinal capillary vessel density alteration associated with retinal vasculitis [34–36]. In the first one, Emre S et al., 2019 conducted an analytical cross-sectional study of 16 patients (32 eyes) with Behcet’s uveitis [36]. They calculated the capillary vessel density up to 1.25 mm from the foveolar centre. The results suggested that the eyes with a history of previous retinal vasculitis had significantly lower foveal capillary vessel density in superficial plexi (20.5% vasculitis vs 29.3% no vasculitis); and deep plexi (21.3% vasculitis vs 31.7% no vasculitis, *p*-value < 0.05, student t-test).

Tian M et al., 2019 [34] conducted an analytical cross-sectional study of 58 patients (93 eyes) with intermediate uveitis (IU). Here, they defined capillary non-perfusion on OCT-A as an area of total and profound capillary loss more than or equal to a quarter of the disc [50]. Capillary non-perfusion was compared between eyes with and without retinal vasculitis using the Chi-squared test. On the widefield montage scan, IU with vasculitis had a higher prevalence of non-perfusion of the superficial capillary plexus (19% vasculitis vs 3% no vasculitis, *p*-value = 0.008); and deep capillary plexus (29% vasculitis vs 7% no vasculitis, *p*-value = 0.008). However, OCT-A 3 × 3 scan did not show any difference in non-perfusion of the superficial capillary plexus (10% vasculitis vs 14% no vasculitis, *p*-value = 0.6) and deep capillary plexus (29% vasculitis vs 18% no vasculitis, *p*-value = 0.3). Foveal avascular zone parameters, including raw length, circularity and size, also differed between IU only and IU with vasculitis (*p*-value < 0.05, ANOVA test). However, the multivariable regression analysis showed these effects were accounted for by epiretinal membrane and cystoid macular oedema rather than the disease entity.

A consecutive analytical cross-sectional study from the same group [35] explored 88 IU patients (164 eyes). Capillary non-perfusion was defined as before, and capillary hypoperfusion was defined as an area of reduced capillary density less that one quarter of the disc [50]. The capillary non-perfusion and hypoperfusion on wide-field montage scans were compared with both the presence of retinal vasculitis and the presence of capillary leakage on fluorescein angiography images. Their result showed that IU with vasculitis had non-perfusion and hypoperfusion in deep capillary plexus and superficial capillary plexus more frequently than IU without vasculitis (Phi and Cramer's V, *p*-value < 0.05); but not choriocapillaris nor choroid. There was no association between capillary leakage on fluorescein angiography and non-perfusion or reduced perfusion in superficial capillary plexus, deep capillary plexus, choriocapillaris or choroid. Furthermore, the study compared the diagnostic accuracy of capillary non-perfusion in OCT-A with the gold standard of capillary dropout and ischaemia finding in fluorescein angiography images. The sensitivity of detection of capillary dropout in superficial capillary plexus and deep capillary plexus were 15% and 24%, while the specificity was 97% and 94%, respectively.

The recent study by Abroug N et al. retrospectively analysed 408 eyes of 284 retinal vasculitis patients [39]. Among these patients, OCT-A was performed in 110 eyes. They found that OCT-A of occlusive retinal vasculitis (50 eyes) showed more frequent areas of capillary nonperfusion or hypoperfusion comparing with non-occlusive retinal vasculitis (60 eyes) (Fischer’s exact test, *p* < 0.001).

#### Assessment of the perivascular retinal thickness

The retrospective case series analysed 12 × 12 mm sweptsource OCT-A scans of 17 patients with retinal vasculitis [46]. These 12 × 12 mm fovea-centred scans allowed the OCT-A machine to image a relatively large area without using a montage image. The authors combined total retinal thickness maps with retinal flow (customised slab between 49 and 149um beneath the internal limiting membrane) in an enface image. They found that the perivascular retinal thickening measured by this method was visually correlated with the leakage/staining on FA imaging (no statistical analysis). There was also a reduction in perivascular retinal thickening with successful treatment in a small subgroup of patients.

## Discussion

Retinal vasculitis can present as part of many different uveitis entities, including infectious, non-infectious with systemic disease, purely ocular with known phenotype or idiopathic [3, 8]. The clinical heterogeneity of retinal vasculitis and the fact that there are no currently standard criteria for diagnosis or classification of the disease can make the assessment of retinal vasculitis challenging [7, 13]. The Standardisation of Uveitis Nomenclature (SUN) working group indicate that more studies are needed to define retinal vasculitis [6], and there is also no consensus on grading its severity. Despite its invasiveness and time taken, fluorescein angiography is still the gold standard and clinical mainstay for diagnosing and assessing retinal vasculitis [10–12] usually in a widefield format [51]. Our systematic review explored alternative imaging to understand OCT findings in retinal vasculitis. We have found that research into OCT, EDI-OCT and OCT-A in retinal vasculitis has predominantly focused on its secondary effects and complications. The OCT findings from imaging inflamed vessels and perivascular tissue has been mostly ignored. One study reported a decrease in perivascular retinal thickness after prednisolone treatment for retinal vasculitis, but in only 3 patients [43]. One case series of 11 patients investigated primary signs of retinal vasculitis in OCT-A [45] but reported none detectable. Another case series utilising swept-source OCT-A reported perivascular retinal thickening visually correlated with vascular leakage/staining in FA imaging but didn’t report their statistic calculation [46]. To the best of our knowledge and through our searching strategy, there is no other study investigating the primary sign of inflammation in or around the retinal vessels in OCT, OCT-EDI or OCT-A. This may in part be because inflamed vessels are frequently located outside the standard field of view of OCT, and certainly a 3 × 3 mm OCT-A.

The secondary effects of retinal vasculitis on the macula are much easier to observe with OCT. These OCT features include cystoid macule oedema (reported in 3 studies) [28, 30, 42], epiretinal membrane (reported in 4 studies) [28, 30, 40, 42], and the disruption in the ellipsoid zone (reported in 2 studies) [40, 41]. These secondary effects are non-specific so could not be used to diagnose retinal vasculitis. However, some of this research suggests unsurprisingly that secondary complications increase the risk of poorer visual prognosis [5, 52]. This evidence supports using OCT to monitor these secondary complications in retinal vasculitis [53, 54].

The thickness of the retina or macula measured by OCT is a potential indicator of retinal inflammation, but again is non-specific and may be due to an inflammatory effect or intraretinal oedema. Four studies used OCT to measure retinal/macular thickness and compared it with the presence of retinal vasculitis or fluorescein angiography leakage score [28, 29, 38, 43]. The results suggest that increased retinal/macular thickness correlates with retinal vasculitis, but it is not clear if this is purely driven by macular oedema. Two studies hypothesised that the increased thickness demonstrates retinal inflammation, and macular oedema could be the late consequence of inflammation [29, 38]. Retinal vasculitis showed some association to increased choroidal thickness measured by OCT-EDI in 4 studies, but not a fifth [31–33, 37, 47]. Two of these studies were in birdshot chorioretinopathy which is disease with a choroidal inflammatory component, so increased choroidal thickness is likely to reflect overall disease activity. An alteration in choroidal thickness was not present in Behcet's uveitis, a disease specifically causing inflammation of the retina and its vasculature [32]. Unless retinal vasculitis is linked to choroidal inflammation (as in birdshot chorioretinopathy) it remains unclear how retinal vasculitis would be linked to an increase in choroidal thickness. It is evident that further research is needed to investigate choroidal thickness parameters in retinal vasculitis in a range of uveities.

In OCT-A, capillary vessel density seems to be a promising tool for assessing occlusive features of retinal vasculitis, although it is currently limited by the small field of view confined to the central macula. One study tried to overcome this with a montage OCT-A with more positive results. Four studies investigated capillary vessel density and non-perfusion using OCT-A and suggested decreased capillary vessel density or more non-perfusion in retinal vasculitis. One study reported the specificity of OCT-A to detect capillary dropout in superficial and deep capillary plexuses was 97% and 94% [35], but the very low sensitivity will mean OCT-A will miss a high proportion with non-perfusion on fluorescein angiography. Capillary vessel density was also explored in a recent meta-analysis in Behcet's patients regardless of retinal vasculitis [55] and showed decreased deep and superficial capillary vessel density in Behcet's disease.

Our study's limitations include limiting studies to those published in English which was a pragmatic approach to the resources available. Otherwise we are confident that our clear and broad search terms will have identified the relevant studies in this area. We only identified a small number of studies which were all retrospective or cross-sectional, investigating different features of the disease, at different stages (inactive or active or previous retinal vasculitis), and with different study designs. This made it impossible to conduct a meta-analysis or other statistical comparison between studies. Moreover, each study used different criteria to diagnose retinal vasculitis and different fluorescein angiography grading systems [28, 29, 32, 38, 44].

There is clearly a large deficit in our knowledge of OCT, EDI-OCT and OCT-A in retinal vasculitis, especially when compared to other retinal diseases. There is evidently a need to investigate the OCT features of inflamed retinal vessels and perivascular retina, and the relationship between retinal vasculitis and structural changes in the macula and choroid, and how they might be used to monitor progressive disease. OCT-A has the potential to provide more information on capillary perfusion in retinal vasculitis but is limited by a narrow field of view to the central macula at present. Swept-source OCT may overcome some of these issues, but further research investigating its findings in retinal vasculitis is awaited.

## Conclusion

In conclusion, our systematic review found a paucity of research into OCT in retinal vasculitis and that it was mostly focused on the secondary effects of retinal vasculitis affecting the macula in specific uveitis entities. There is no evidence to suggest that in the near future current OCT technologies are likely to supersede fluorescein angiography in identifying active retinal vasculitis and associated non-perfusion, or grading its severity.

## Supplementary Information

**Additional file 1.**

## Data Availability

All data are present in the manuscript.

## Abbreviations

FA: Fluorescein angiography
IU: Intermediate uveitis
OCT: Optical coherence tomography
OCT-A: OCT angiography
OCT-EDI: Enhanced-depth imaging OCT
SLE: Systemic lupus erythematosus
