# Supplementary material for: A systematic review of OCT and OCT angiography in retinal vasculitis
Source: J Ophthalmic Inflamm Infect. 2023 Jan 30;13:1. doi: 10.1186/s12348-023-00327-4 (PMC9886715; doi:10.1186/s12348-023-00327-4)
Supplement: Supplementary file 1 — Additional file 1. [file 12348_2023_327_MOESM1_ESM.docx]

| The JBI Critical Appraisal Checklist for Case Series  Comparison between optical coherence tomography angiography and fluorescein angiography findings in retinal vasculitis | | | | |
| --- | --- | --- | --- | --- |
| Major Components | Response options | | | |
| 1. Were there clear criteria for inclusion in the case series? | Yes | No | Unclear | Not applicable |
| 2. Was the condition measured in a standard, reliable way for all participants included in the case series? | Yes | No | Unclear | Not applicable |
| 3. Were valid methods used for identification of the condition for all participants included in the case series? | Yes | No | Unclear | Not applicable |
| 4. Did the case series have consecutive inclusion of participants? | Yes | No | Unclear | Not applicable |
| 5. Did the case series have complete inclusion of participants? | Yes | No | Unclear | Not applicable |
| 6. Was there clear reporting of the demographics of the participants in the study? | Yes | No | Unclear | Not applicable |
| 7. Was there clear reporting of clinical information of the participants? | Yes | No | Unclear | Not applicable |
| 8. Were the outcomes or follow up results of cases clearly reported? | Yes | No | Unclear | Not applicable |
| 9. Was there clear reporting of the presenting site(s)/clinic(s) demographic information? | Yes | No | Unclear | Not applicable |
| 10. Was statistical analysis appropriate? | Yes | No | Unclear | Not applicable |
| Overall appraisal: Include 🗹 Exclude □ Seek further info □ | | | | |

| The JBI Critical Appraisal Checklist for Case Series  Correlation Between Clinical Signs and Optical Coherence Tomography With Enhanced Depth Imaging Findings in Patients With Birdshot Chorioretinopathy | | | | |
| --- | --- | --- | --- | --- |
| Major Components | Response options | | | |
| 1. Were there clear criteria for inclusion in the case series? | Yes | No | Unclear | Not applicable |
| 2. Was the condition measured in a standard, reliable way for all participants included in the case series? | Yes | No | Unclear | Not applicable |
| 3. Were valid methods used for identification of the condition for all participants included in the case series? | Yes | No | Unclear | Not applicable |
| 4. Did the case series have consecutive inclusion of participants? | Yes | No | Unclear | Not applicable |
| 5. Did the case series have complete inclusion of participants? | Yes | No | Unclear | Not applicable |
| 6. Was there clear reporting of the demographics of the participants in the study? | Yes | No | Unclear | Not applicable |
| 7. Was there clear reporting of clinical information of the participants? | Yes | No | Unclear | Not applicable |
| 8. Were the outcomes or follow up results of cases clearly reported? | Yes | No | Unclear | Not applicable |
| 9. Was there clear reporting of the presenting site(s)/clinic(s) demographic information? | Yes | No | Unclear | Not applicable |
| 10. Was statistical analysis appropriate? | Yes | No | Unclear | Not applicable |
| Overall appraisal: Include 🗹 Exclude □ Seek further info □ | | | | |

| The JBI Critical Appraisal Checklist for analytical cross-sectional study  Choroidal findings in eyes with birdshot chorioretinitis using enhanced-depth optical coherence tomography. | | | | |
| --- | --- | --- | --- | --- |
| Major Components | Response options | | | |
| 1. Were the criteria for inclusion in the sample clearly defined? | Yes | No | Unclear | Not applicable |
| 2. Were the study subjects and the setting described in detail? | Yes | No | Unclear | Not applicable |
| 3. Was the exposure measured in a valid and reliable way? | Yes | No | Unclear | Not applicable |
| 4. Were objective, standard criteria used for measurement of the condition? | Yes | No | Unclear | Not applicable |
| 5. Were confounding factors identified? | Yes | No | Unclear | Not applicable |
| 6. Were strategies to deal with confounding factors stated? | Yes | No | Unclear | Not applicable |
| 7. Were the outcomes measured in a valid and reliable way? | Yes | No | Unclear | Not applicable |
| 8. Was appropriate statistical analysis used? | Yes | No | Unclear | Not applicable |
| Overall appraisal: Include 🗹 Exclude □ Seek further info □ | | | | |

| The JBI Critical Appraisal Checklist for analytical cross-sectional study  Choroidal Structural Changes in Patients with Birdshot Chorioretinopathy. | | | | |
| --- | --- | --- | --- | --- |
| Major Components | Response options | | | |
| 1. Were the criteria for inclusion in the sample clearly defined? | Yes | No | Unclear | Not applicable |
| 2. Were the study subjects and the setting described in detail? | Yes | No | Unclear | Not applicable |
| 3. Was the exposure measured in a valid and reliable way? | Yes | No | Unclear | Not applicable |
| 4. Were objective, standard criteria used for measurement of the condition? | Yes | No | Unclear | Not applicable |
| 5. Were confounding factors identified? | Yes | No | Unclear | Not applicable |
| 6. Were strategies to deal with confounding factors stated? | Yes | No | Unclear | Not applicable |
| 7. Were the outcomes measured in a valid and reliable way? | Yes | No | Unclear | Not applicable |
| 8. Was appropriate statistical analysis used? | Yes | No | Unclear | Not applicable |
| Overall appraisal: Include 🗹 Exclude □ Seek further info □ | | | | |

| The JBI Critical Appraisal Checklist for analytical cross-sectional study  Optical coherence tomography angiography findings in Behcet patients. | | | | |
| --- | --- | --- | --- | --- |
| Major Components | Response options | | | |
| 1. Were the criteria for inclusion in the sample clearly defined? | Yes | No | Unclear | Not applicable |
| 2. Were the study subjects and the setting described in detail? | Yes | No | Unclear | Not applicable |
| 3. Was the exposure measured in a valid and reliable way? | Yes | No | Unclear | Not applicable |
| 4. Were objective, standard criteria used for measurement of the condition? | Yes | No | Unclear | Not applicable |
| 5. Were confounding factors identified? | Yes | No | Unclear | Not applicable |
| 6. Were strategies to deal with confounding factors stated? | Yes | No | Unclear | Not applicable |
| 7. Were the outcomes measured in a valid and reliable way? | Yes | No | Unclear | Not applicable |
| 8. Was appropriate statistical analysis used? | Yes | No | Unclear | Not applicable |
| Overall appraisal: Include 🗹 Exclude □ Seek further info □ | | | | |

| The JBI Critical Appraisal Checklist for Case Series  Spectral domain optical coherence tomography evaluation of macular changes in Eales disease. | | | | |
| --- | --- | --- | --- | --- |
| Major Components | Response options | | | |
| 1. Were there clear criteria for inclusion in the case series? | Yes | No | Unclear | Not applicable |
| 2. Was the condition measured in a standard, reliable way for all participants included in the case series? | Yes | No | Unclear | Not applicable |
| 3. Were valid methods used for identification of the condition for all participants included in the case series? | Yes | No | Unclear | Not applicable |
| 4. Did the case series have consecutive inclusion of participants? | Yes | No | Unclear | Not applicable |
| 5. Did the case series have complete inclusion of participants? | Yes | No | Unclear | Not applicable |
| 6. Was there clear reporting of the demographics of the participants in the study? | Yes | No | Unclear | Not applicable |
| 7. Was there clear reporting of clinical information of the participants? | Yes | No | Unclear | Not applicable |
| 8. Were the outcomes or follow up results of cases clearly reported? | Yes | No | Unclear | Not applicable |
| 9. Was there clear reporting of the presenting site(s)/clinic(s) demographic information? | Yes | No | Unclear | Not applicable |
| 10. Was statistical analysis appropriate? | Yes | No | Unclear | Not applicable |
| Overall appraisal: Include 🗹 Exclude □ Seek further info □ | | | | |

| The JBI Critical Appraisal Checklist for analytical cross-sectional study  Quantitative Analysis of Peripheral Vasculitis, Ischemia, and Vascular Leakage in Uveitis Using Ultra-Widefield Fluorescein Angiography. | | | | |
| --- | --- | --- | --- | --- |
| Major Components | Response options | | | |
| 1. Were the criteria for inclusion in the sample clearly defined? | Yes | No | Unclear | Not applicable |
| 2. Were the study subjects and the setting described in detail? | Yes | No | Unclear | Not applicable |
| 3. Was the exposure measured in a valid and reliable way? | Yes | No | Unclear | Not applicable |
| 4. Were objective, standard criteria used for measurement of the condition? | Yes | No | Unclear | Not applicable |
| 5. Were confounding factors identified? | Yes | No | Unclear | Not applicable |
| 6. Were strategies to deal with confounding factors stated? | Yes | No | Unclear | Not applicable |
| 7. Were the outcomes measured in a valid and reliable way? | Yes | No | Unclear | Not applicable |
| 8. Was appropriate statistical analysis used? | Yes | No | Unclear | Not applicable |
| Overall appraisal: Include 🗹 Exclude □ Seek further info □ | | | | |

| The JBI Critical Appraisal Checklist for analytical cross-sectional study  QUANTITATIVE ANALYSIS OF STRUCTURAL ALTERATIONS IN THE CHOROID OF PATIENTS WITH ACTIVE BEHÇET UVEITIS | | | | |
| --- | --- | --- | --- | --- |
| Major Components | Response options | | | |
| 1. Were the criteria for inclusion in the sample clearly defined? | Yes | No | Unclear | Not applicable |
| 2. Were the study subjects and the setting described in detail? | Yes | No | Unclear | Not applicable |
| 3. Was the exposure measured in a valid and reliable way? | Yes | No | Unclear | Not applicable |
| 4. Were objective, standard criteria used for measurement of the condition? | Yes | No | Unclear | Not applicable |
| 5. Were confounding factors identified? | Yes | No | Unclear | Not applicable |
| 6. Were strategies to deal with confounding factors stated? | Yes | No | Unclear | Not applicable |
| 7. Were the outcomes measured in a valid and reliable way? | Yes | No | Unclear | Not applicable |
| 8. Was appropriate statistical analysis used? | Yes | No | Unclear | Not applicable |
| Overall appraisal: Include 🗹 Exclude □ Seek further info □ | | | | |

| The JBI Critical Appraisal Checklist for Case Series  Non-invasive method of monitoring retinal vasculitis in patients with birdshot chorioretinopathy using optical coherence tomography. | | | | |
| --- | --- | --- | --- | --- |
| Major Components | Response options | | | |
| 1. Were there clear criteria for inclusion in the case series? | Yes | No | Unclear | Not applicable |
| 2. Was the condition measured in a standard, reliable way for all participants included in the case series? | Yes | No | Unclear | Not applicable |
| 3. Were valid methods used for identification of the condition for all participants included in the case series? | Yes | No | Unclear | Not applicable |
| 4. Did the case series have consecutive inclusion of participants? | Yes | No | Unclear | Not applicable |
| 5. Did the case series have complete inclusion of participants? | Yes | No | Unclear | Not applicable |
| 6. Was there clear reporting of the demographics of the participants in the study? | Yes | No | Unclear | Not applicable |
| 7. Was there clear reporting of clinical information of the participants? | Yes | No | Unclear | Not applicable |
| 8. Were the outcomes or follow up results of cases clearly reported? | Yes | No | Unclear | Not applicable |
| 9. Was there clear reporting of the presenting site(s)/clinic(s) demographic information? | Yes | No | Unclear | Not applicable |
| 10. Was statistical analysis appropriate? | Yes | No | Unclear | Not applicable |
| Overall appraisal: Include 🗹 Exclude □ Seek further info □ | | | | |

| The JBI Critical Appraisal Checklist for case-control study  Choroidal thickness alterations in idiopathic acute retinal vasculitis. | | | | |
| --- | --- | --- | --- | --- |
| Major Components | Response options | | | |
| 1. Were the groups comparable other than the presence of disease in cases or the absence of disease in controls? | Yes | No | Unclear | Not applicable |
| 2. Were cases and controls matched appropriately? | Yes | No | Unclear | Not applicable |
| 3. Were the same criteria used for identification of cases and controls? | Yes | No | Unclear | Not applicable |
| 4. Was exposure measured in a standard, valid and reliable way? | Yes | No | Unclear | Not applicable |
| 5. Was exposure measured in the same way for cases and controls? | Yes | No | Unclear | Not applicable |
| 6. Were confounding factors identified? | Yes | No | Unclear | Not applicable |
| 7. Were strategies to deal with confounding factors stated? | Yes | No | Unclear | Not applicable |
| 8. Were outcomes assessed in a standard, valid and reliable way for cases and controls? | Yes | No | Unclear | Not applicable |
| 9. Was the exposure period of interest long enough to be meaningful? | Yes | No | Unclear | Not applicable |
| 10. Was appropriate statistical analysis used? | Yes | No | Unclear | Not applicable |
| Overall appraisal: Include 🗹 Exclude □ Seek further info □ | | | | |

| The JBI Critical Appraisal Checklist for analytical cross-sectional study  Visual outcome and poor prognostic factors in isolated idiopathic retinal vasculitis. | | | | |
| --- | --- | --- | --- | --- |
| Major Components | Response options | | | |
| 1. Were the criteria for inclusion in the sample clearly defined? | Yes | No | Unclear | Not applicable |
| 2. Were the study subjects and the setting described in detail? | Yes | No | Unclear | Not applicable |
| 3. Was the exposure measured in a valid and reliable way? | Yes | No | Unclear | Not applicable |
| 4. Were objective, standard criteria used for measurement of the condition? | Yes | No | Unclear | Not applicable |
| 5. Were confounding factors identified? | Yes | No | Unclear | Not applicable |
| 6. Were strategies to deal with confounding factors stated? | Yes | No | Unclear | Not applicable |
| 7. Were the outcomes measured in a valid and reliable way? | Yes | No | Unclear | Not applicable |
| 8. Was appropriate statistical analysis used? | Yes | No | Unclear | Not applicable |
| Overall appraisal: Include 🗹 Exclude □ Seek further info □ | | | | |

| The JBI Critical Appraisal Checklist for analytical cross-sectional study  Longitudinal cohort study of patients with birdshot chorioretinopathy. III. Macular imaging at baseline. | | | | |
| --- | --- | --- | --- | --- |
| Major Components | Response options | | | |
| 1. Were the criteria for inclusion in the sample clearly defined? | Yes | No | Unclear | Not applicable |
| 2. Were the study subjects and the setting described in detail? | Yes | No | Unclear | Not applicable |
| 3. Was the exposure measured in a valid and reliable way? | Yes | No | Unclear | Not applicable |
| 4. Were objective, standard criteria used for measurement of the condition? | Yes | No | Unclear | Not applicable |
| 5. Were confounding factors identified? | Yes | No | Unclear | Not applicable |
| 6. Were strategies to deal with confounding factors stated? | Yes | No | Unclear | Not applicable |
| 7. Were the outcomes measured in a valid and reliable way? | Yes | No | Unclear | Not applicable |
| 8. Was appropriate statistical analysis used? | Yes | No | Unclear | Not applicable |
| Overall appraisal: Include 🗹 Exclude □ Seek further info □ | | | | |

| The JBI Critical Appraisal Checklist for analytical cross-sectional study  Association between subfoveal choroidal thickness and leakage site on fluorescein angiography in Behcet's uveitis. | | | | |
| --- | --- | --- | --- | --- |
| Major Components | Response options | | | |
| 1. Were the criteria for inclusion in the sample clearly defined? | Yes | No | Unclear | Not applicable |
| 2. Were the study subjects and the setting described in detail? | Yes | No | Unclear | Not applicable |
| 3. Was the exposure measured in a valid and reliable way? | Yes | No | Unclear | Not applicable |
| 4. Were objective, standard criteria used for measurement of the condition? | Yes | No | Unclear | Not applicable |
| 5. Were confounding factors identified? | Yes | No | Unclear | Not applicable |
| 6. Were strategies to deal with confounding factors stated? | Yes | No | Unclear | Not applicable |
| 7. Were the outcomes measured in a valid and reliable way? | Yes | No | Unclear | Not applicable |
| 8. Was appropriate statistical analysis used? | Yes | No | Unclear | Not applicable |
| Overall appraisal: Include 🗹 Exclude □ Seek further info □ | | | | |

| The JBI Critical Appraisal Checklist for Case Series  Multimodal imaging of the disease progression of birdshot chorioretinopathy. | | | | |
| --- | --- | --- | --- | --- |
| Major Components | Response options | | | |
| 1. Were there clear criteria for inclusion in the case series? | Yes | No | Unclear | Not applicable |
| 2. Was the condition measured in a standard, reliable way for all participants included in the case series? | Yes | No | Unclear | Not applicable |
| 3. Were valid methods used for identification of the condition for all participants included in the case series? | Yes | No | Unclear | Not applicable |
| 4. Did the case series have consecutive inclusion of participants? | Yes | No | Unclear | Not applicable |
| 5. Did the case series have complete inclusion of participants? | Yes | No | Unclear | Not applicable |
| 6. Was there clear reporting of the demographics of the participants in the study? | Yes | No | Unclear | Not applicable |
| 7. Was there clear reporting of clinical information of the participants? | Yes | No | Unclear | Not applicable |
| 8. Were the outcomes or follow up results of cases clearly reported? | Yes | No | Unclear | Not applicable |
| 9. Was there clear reporting of the presenting site(s)/clinic(s) demographic information? | Yes | No | Unclear | Not applicable |
| 10. Was statistical analysis appropriate? | Yes | No | Unclear | Not applicable |
| Overall appraisal: Include 🗹 Exclude □ Seek further info □ | | | | |

| The JBI Critical Appraisal Checklist for analytical cross-sectional study  Evaluation of vascular changes in intermediate uveitis and retinal vasculitis using swept-source wide-field optical coherence tomography angiography. | | | | |
| --- | --- | --- | --- | --- |
| Major Components | Response options | | | |
| 1. Were the criteria for inclusion in the sample clearly defined? | Yes | No | Unclear | Not applicable |
| 2. Were the study subjects and the setting described in detail? | Yes | No | Unclear | Not applicable |
| 3. Was the exposure measured in a valid and reliable way? | Yes | No | Unclear | Not applicable |
| 4. Were objective, standard criteria used for measurement of the condition? | Yes | No | Unclear | Not applicable |
| 5. Were confounding factors identified? | Yes | No | Unclear | Not applicable |
| 6. Were strategies to deal with confounding factors stated? | Yes | No | Unclear | Not applicable |
| 7. Were the outcomes measured in a valid and reliable way? | Yes | No | Unclear | Not applicable |
| 8. Was appropriate statistical analysis used? | Yes | No | Unclear | Not applicable |
| Overall appraisal: Include 🗹 Exclude □ Seek further info □ | | | | |

| The JBI Critical Appraisal Checklist for analytical cross-sectional study  Swept-source optical coherence tomography angiography reveals vascular changes in intermediate uveitis. | | | | |
| --- | --- | --- | --- | --- |
| Major Components | Response options | | | |
| 1. Were the criteria for inclusion in the sample clearly defined? | Yes | No | Unclear | Not applicable |
| 2. Were the study subjects and the setting described in detail? | Yes | No | Unclear | Not applicable |
| 3. Was the exposure measured in a valid and reliable way? | Yes | No | Unclear | Not applicable |
| 4. Were objective, standard criteria used for measurement of the condition? | Yes | No | Unclear | Not applicable |
| 5. Were confounding factors identified? | Yes | No | Unclear | Not applicable |
| 6. Were strategies to deal with confounding factors stated? | Yes | No | Unclear | Not applicable |
| 7. Were the outcomes measured in a valid and reliable way? | Yes | No | Unclear | Not applicable |
| 8. Was appropriate statistical analysis used? | Yes | No | Unclear | Not applicable |
| Overall appraisal: Include 🗹 Exclude □ Seek further info □ | | | | |

| The JBI Critical Appraisal Checklist for analytical cross-sectional study  Peripapillary optical coherence tomography as an alternative to fluorescein angiography for monitoring Behcet's retinal vasculitis. | | | | |
| --- | --- | --- | --- | --- |
| Major Components | Response options | | | |
| 1. Were the criteria for inclusion in the sample clearly defined? | Yes | No | Unclear | Not applicable |
| 2. Were the study subjects and the setting described in detail? | Yes | No | Unclear | Not applicable |
| 3. Was the exposure measured in a valid and reliable way? | Yes | No | Unclear | Not applicable |
| 4. Were objective, standard criteria used for measurement of the condition? | Yes | No | Unclear | Not applicable |
| 5. Were confounding factors identified? | Yes | No | Unclear | Not applicable |
| 6. Were strategies to deal with confounding factors stated? | Yes | No | Unclear | Not applicable |
| 7. Were the outcomes measured in a valid and reliable way? | Yes | No | Unclear | Not applicable |
| 8. Was appropriate statistical analysis used? | Yes | No | Unclear | Not applicable |
| Overall appraisal: Include 🗹 Exclude □ Seek further info □ | | | | |

| The JBI Critical Appraisal Checklist for Case Series  A Novel Method to Detect and Monitor Retinal Vasculitis Using Swept-Source OCT Angiography. | | | | |
| --- | --- | --- | --- | --- |
| Major Components | Response options | | | |
| 1. Were there clear criteria for inclusion in the case series? | Yes | No | Unclear | Not applicable |
| 2. Was the condition measured in a standard, reliable way for all participants included in the case series? | Yes | No | Unclear | Not applicable |
| 3. Were valid methods used for identification of the condition for all participants included in the case series? | Yes | No | Unclear | Not applicable |
| 4. Did the case series have consecutive inclusion of participants? | Yes | No | Unclear | Not applicable |
| 5. Did the case series have complete inclusion of participants? | Yes | No | Unclear | Not applicable |
| 6. Was there clear reporting of the demographics of the participants in the study? | Yes | No | Unclear | Not applicable |
| 7. Was there clear reporting of clinical information of the participants? | Yes | No | Unclear | Not applicable |
| 8. Were the outcomes or follow up results of cases clearly reported? | Yes | No | Unclear | Not applicable |
| 9. Was there clear reporting of the presenting site(s)/clinic(s) demographic information? | Yes | No | Unclear | Not applicable |
| 10. Was statistical analysis appropriate? | Yes | No | Unclear | Not applicable |
| Overall appraisal: Include 🗹 Exclude □ Seek further info □ | | | | |

| The JBI Critical Appraisal Checklist for analytical cross-sectional study  A Comparative Study between Occlusive and Non-occlusive Retinal Vasculitis: Data from a Referral Center in Tunisia. | | | | |
| --- | --- | --- | --- | --- |
| Major Components | Response options | | | |
| 1. Were the criteria for inclusion in the sample clearly defined? | Yes | No | Unclear | Not applicable |
| 2. Were the study subjects and the setting described in detail? | Yes | No | Unclear | Not applicable |
| 3. Was the exposure measured in a valid and reliable way? | Yes | No | Unclear | Not applicable |
| 4. Were objective, standard criteria used for measurement of the condition? | Yes | No | Unclear | Not applicable |
| 5. Were confounding factors identified? | Yes | No | Unclear | Not applicable |
| 6. Were strategies to deal with confounding factors stated? | Yes | No | Unclear | Not applicable |
| 7. Were the outcomes measured in a valid and reliable way? | Yes | No | Unclear | Not applicable |
| 8. Was appropriate statistical analysis used? | Yes | No | Unclear | Not applicable |
| Overall appraisal: Include 🗹 Exclude □ Seek further info □ | | | | |

| The JBI Critical Appraisal Checklist for analytical cross-sectional study  ISOLATED RETINAL VASCULITIS: Prognostic Factors and Expanding the Role of Immunosuppressive Treatment in Retinal Vasculitis Associated With Positive QuantiFERON-TB Gold Test. | | | | |
| --- | --- | --- | --- | --- |
| Major Components | Response options | | | |
| 1. Were the criteria for inclusion in the sample clearly defined? | Yes | No | Unclear | Not applicable |
| 2. Were the study subjects and the setting described in detail? | Yes | No | Unclear | Not applicable |
| 3. Was the exposure measured in a valid and reliable way? | Yes | No | Unclear | Not applicable |
| 4. Were objective, standard criteria used for measurement of the condition? | Yes | No | Unclear | Not applicable |
| 5. Were confounding factors identified? | Yes | No | Unclear | Not applicable |
| 6. Were strategies to deal with confounding factors stated? | Yes | No | Unclear | Not applicable |
| 7. Were the outcomes measured in a valid and reliable way? | Yes | No | Unclear | Not applicable |
| 8. Was appropriate statistical analysis used? | Yes | No | Unclear | Not applicable |
| Overall appraisal: Include 🗹 Exclude □ Seek further info □ | | | | |
